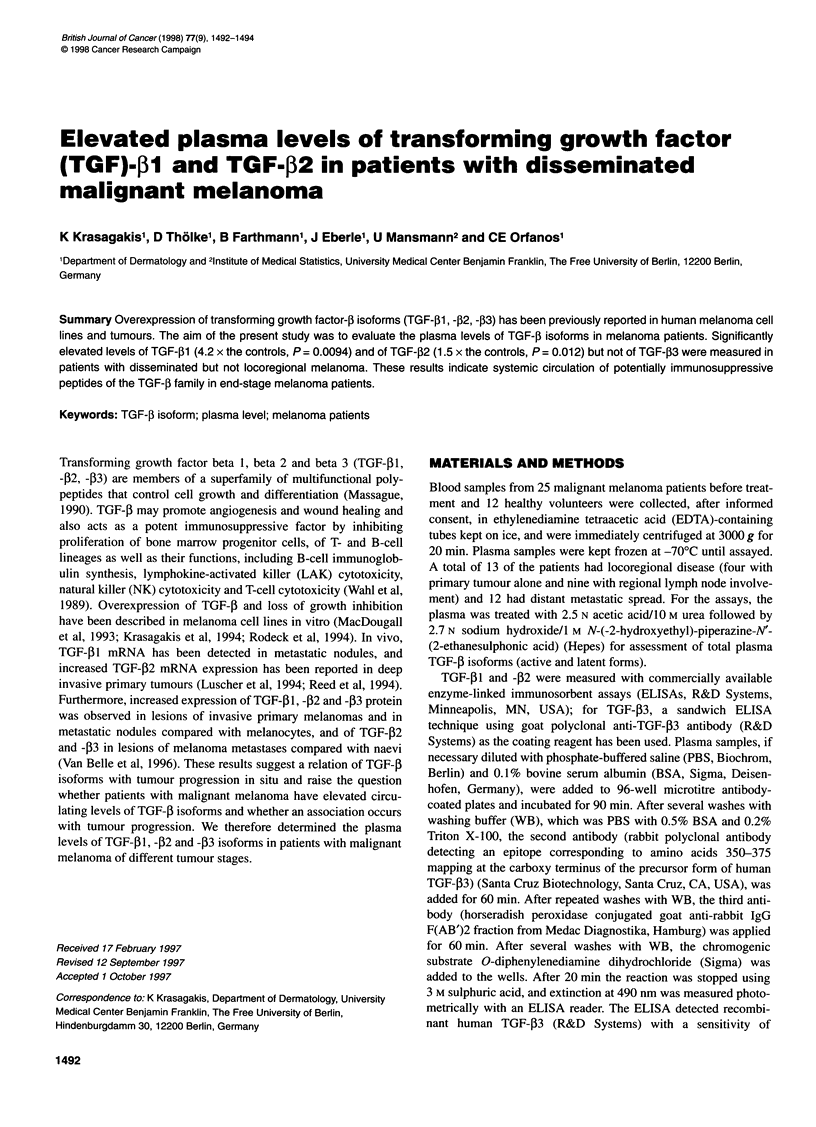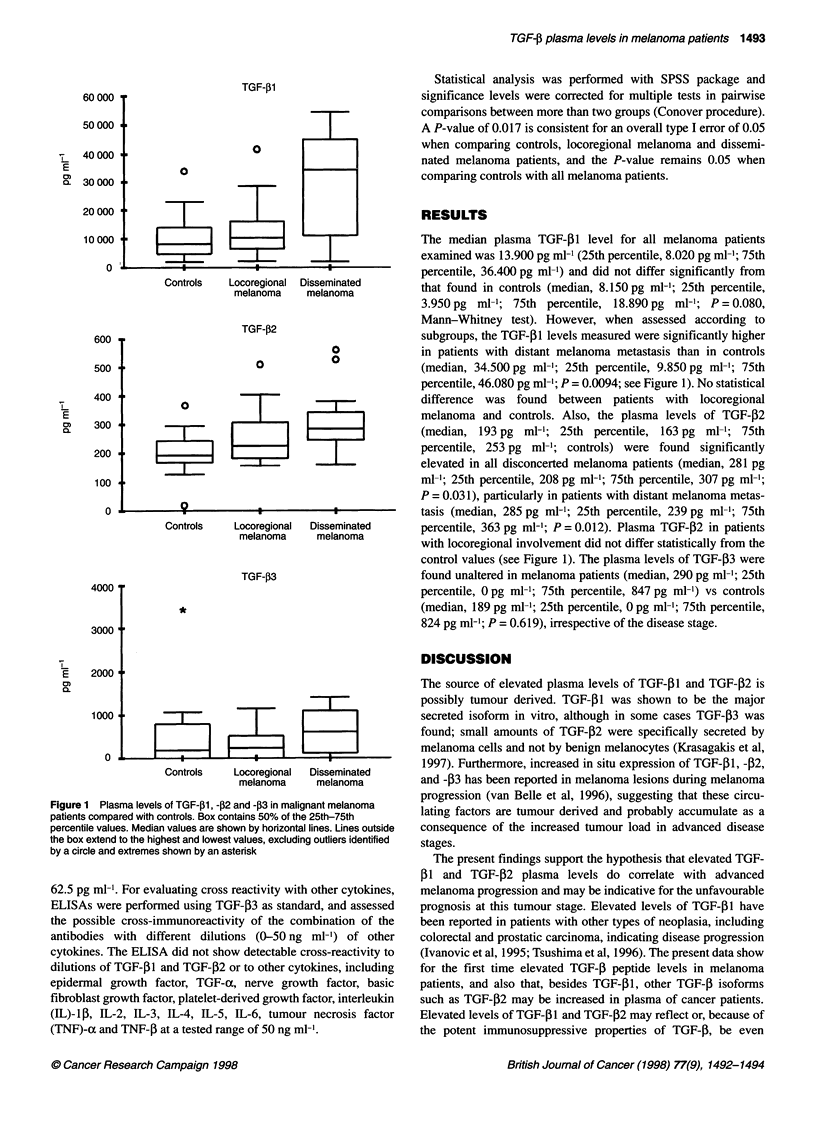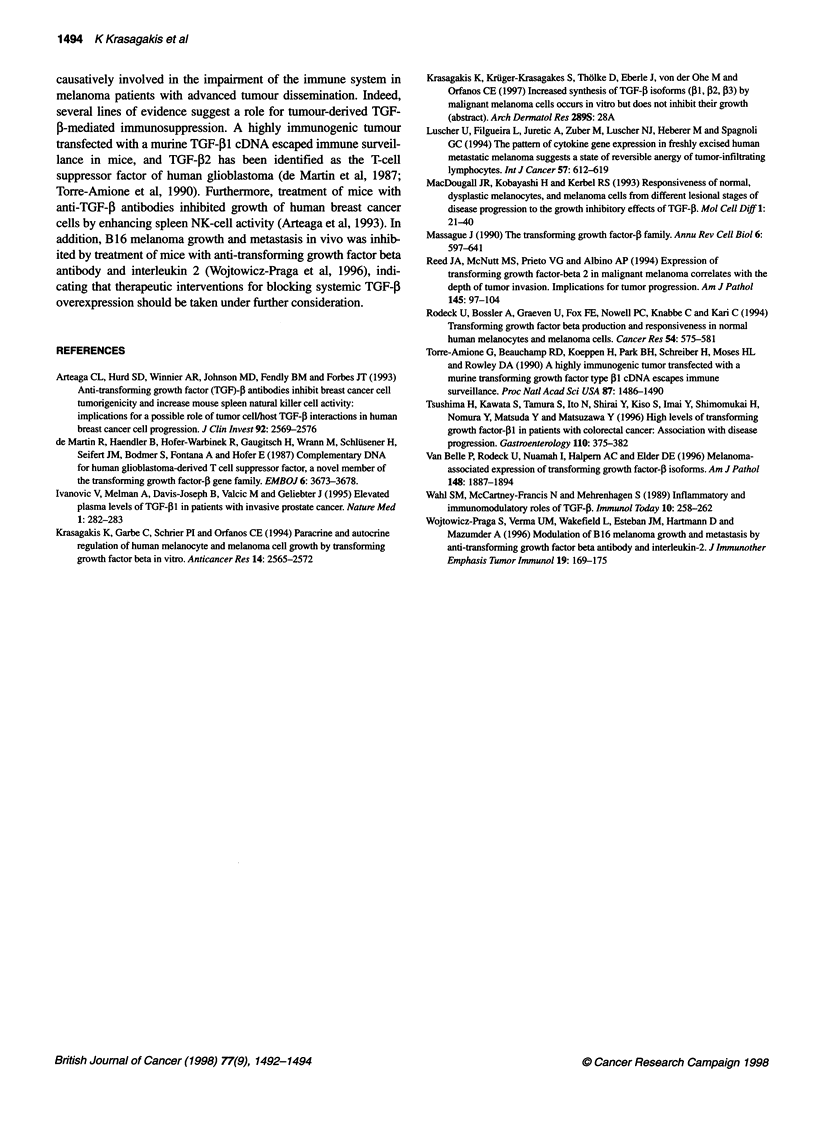# Elevated plasma levels of transforming growth factor (TGF)-beta1 and TGF-beta2 in patients with disseminated malignant melanoma.

**DOI:** 10.1038/bjc.1998.245

**Published:** 1998-05

**Authors:** K. Krasagakis, D. ThÃ¶lke, B. Farthmann, J. Eberle, U. Mansmann, C. E. Orfanos

**Affiliations:** Department of Dermatology, University Medical Center Benjamin Franklin, The Free University of Berlin, Germany.

## Abstract

Overexpression of transforming growth factor-beta isoforms (TGF-beta1, -beta2, -beta3) has been previously reported in human melanoma cell lines and tumours. The aim of the present study was to evaluate the plasma levels of TGF-beta isoforms in melanoma patients. Significantly elevated levels of TGF-beta1 (4.2 x the controls, P = 0.0094) and of TGF-beta2 (1.5 x the controls, P = 0.012) but not of TGF-beta3 were measured in patients with disseminated but not locoregional melanoma. These results indicate systemic circulation of potentially immunosuppressive peptides of the TGF-beta family in end-stage melanoma patients.


British Journal of Cancer (1998) 77(9), 1492–1494
© 1998 Cancer Research Campaign

### Elevated plasma levels of transforming growth factor (TGF)-β1 and TGF-β2 in patients with disseminated malignant melanoma

**K Krasagakis[1], D Thölke[1], B Farthmann[1], J Eberle[1], U Mansmann[2] and CE Orfanos[1]**

[1]Department of Dermatology and [2]Institute of Medical Statistics, University Medical Center Benjamin Franklin, The Free University of Berlin, 12200 Berlin, Germany

**Summary** Overexpression of transforming growth factor-β isoforms (TGF-β1, -β2, -β3) has been previously reported in human melanoma cell lines and tumours. The aim of the present study was to evaluate the plasma levels of TGF-β isoforms in melanoma patients. Significantly elevated levels of TGF-β1 (4.2 × the controls, $P = 0.0094$) and of TGF-β2 (1.5 × the controls, $P = 0.012$) but not of TGF-β3 were measured in patients with disseminated but not locoregional melanoma. These results indicate systemic circulation of potentially immunosuppressive peptides of the TGF-β family in end-stage melanoma patients.

**Keywords:** TGF-β isoform; plasma level; melanoma patients

Transforming growth factor beta 1, beta 2 and beta 3 (TGF-β1, -β2, -β3) are members of a superfamily of multifunctional polypeptides that control cell growth and differentiation (Massague, 1990). TGF-β may promote angiogenesis and wound healing and also acts as a potent immunosuppressive factor by inhibiting proliferation of bone marrow progenitor cells, of T- and B-cell lineages as well as their functions, including B-cell immunoglobulin synthesis, lymphokine-activated killer (LAK) cytotoxicity, natural killer (NK) cytotoxicity and T-cell cytotoxicity (Wahl et al, 1989). Overexpression of TGF-β and loss of growth inhibition have been described in melanoma cell lines in vitro (MacDougall et al, 1993; Krasagakis et al, 1994; Rodeck et al, 1994). In vivo, TGF-β1 mRNA has been detected in metastatic nodules, and increased TGF-β2 mRNA expression has been reported in deep invasive primary tumours (Luscher et al, 1994; Reed et al, 1994). Furthermore, increased expression of TGF-β1, -β2 and -β3 protein was observed in lesions of invasive primary melanomas and in metastatic nodules compared with melanocytes, and of TGF-β2 and -β3 in lesions of melanoma metastases compared with naevi (Van Belle et al, 1996). These results suggest a relation of TGF-β isoforms with tumour progression in situ and raise the question whether patients with malignant melanoma have elevated circulating levels of TGF-β isoforms and whether an association occurs with tumour progression. We therefore determined the plasma levels of TGF-β1, -β2 and -β3 isoforms in patients with malignant melanoma of different tumour stages.

*Received 17 February 1997*
*Revised 12 September 1997*
*Accepted 1 October 1997*

*Correspondence to:* K Krasagakis, Department of Dermatology, University Medical Center Benjamin Franklin, The Free University of Berlin, Hindenburgdamm 30, 12200 Berlin, Germany

### MATERIALS AND METHODS

Blood samples from 25 malignant melanoma patients before treatment and 12 healthy volunteers were collected, after informed consent, in ethylenediamine tetraacetic acid (EDTA)-containing tubes kept on ice, and were immediately centrifuged at 3000 ***g*** for 20 min. Plasma samples were kept frozen at –70°C until assayed. A total of 13 of the patients had locoregional disease (four with primary tumour alone and nine with regional lymph node involvement) and 12 had distant metastatic spread. For the assays, the plasma was treated with 2.5 N acetic acid/10 M urea followed by 2.7 N sodium hydroxide/1 M *N*-(-2-hydroxyethyl)-piperazine-*N*′-(2-ethanesulphonic acid) (Hepes) for assessment of total plasma TGF-β isoforms (active and latent forms).

TGF-β1 and -β2 were measured with commercially available enzyme-linked immunosorbent assays (ELISAs, R&D Systems, Minneapolis, MN, USA); for TGF-β3, a sandwich ELISA technique using goat polyclonal anti-TGF-β3 antibody (R&D Systems) as the coating reagent has been used. Plasma samples, if necessary diluted with phosphate-buffered saline (PBS, Biochrom, Berlin) and 0.1% bovine serum albumin (BSA, Sigma, Deisenhofen, Germany), were added to 96-well microtitre antibody-coated plates and incubated for 90 min. After several washes with washing buffer (WB), which was PBS with 0.5% BSA and 0.2% Triton X-100, the second antibody (rabbit polyclonal antibody detecting an epitope corresponding to amino acids 350–375 mapping at the carboxy terminus of the precursor form of human TGF-β3) (Santa Cruz Biotechnology, Santa Cruz, CA, USA), was added for 60 min. After repeated washes with WB, the third antibody (horseradish peroxidase conjugated goat anti-rabbit IgG F(AB′)2 fraction from Medac Diagnostika, Hamburg) was applied for 60 min. After several washes with WB, the chromogenic substrate *O*-diphenylenediamine dihydrochloride (Sigma) was added to the wells. After 20 min the reaction was stopped using 3 M sulphuric acid, and extinction at 490 nm was measured photometrically with an ELISA reader. The ELISA detected recombinant human TGF-β3 (R&D Systems) with a sensitivity of

**Figure 1** Plasma levels of TGF-β1, -β2 and -β3 in malignant melanoma patients compared with controls. Box contains 50% of the 25th–75th percentile values. Median values are shown by horizontal lines. Lines outside the box extend to the highest and lowest values, excluding outliers identified by a circle and extremes shown by an asterisk

62.5 pg ml$^{-1}$. For evaluating cross reactivity with other cytokines, ELISAs were performed using TGF-β3 as standard, and assessed the possible cross-immunoreactivity of the combination of the antibodies with different dilutions (0–50 ng ml$^{-1}$) of other cytokines. The ELISA did not show detectable cross-reactivity to dilutions of TGF-β1 and TGF-β2 or to other cytokines, including epidermal growth factor, TGF-α, nerve growth factor, basic fibroblast growth factor, platelet-derived growth factor, interleukin (IL)-1β, IL-2, IL-3, IL-4, IL-5, IL-6, tumour necrosis factor (TNF)-α and TNF-β at a tested range of 50 ng ml$^{-1}$.

Statistical analysis was performed with SPSS package and significance levels were corrected for multiple tests in pairwise comparisons between more than two groups (Conover procedure). A *P*-value of 0.017 is consistent for an overall type I error of 0.05 when comparing controls, locoregional melanoma and disseminated melanoma patients, and the *P*-value remains 0.05 when comparing controls with all melanoma patients.

### RESULTS

The median plasma TGF-β1 level for all melanoma patients examined was 13.900 pg ml$^{-1}$ (25th percentile, 8.020 pg ml$^{-1}$; 75th percentile, 36.400 pg ml$^{-1}$) and did not differ significantly from that found in controls (median, 8.150 pg ml$^{-1}$; 25th percentile, 3.950 pg ml$^{-1}$; 75th percentile, 18.890 pg ml$^{-1}$; $P = 0.080$, Mann–Whitney test). However, when assessed according to subgroups, the TGF-β1 levels measured were significantly higher in patients with distant melanoma metastasis than in controls (median, 34.500 pg ml$^{-1}$; 25th percentile, 9.850 pg ml$^{-1}$; 75th percentile, 46.080 pg ml$^{-1}$; $P = 0.0094$; see Figure 1). No statistical difference was found between patients with locoregional melanoma and controls. Also, the plasma levels of TGF-β2 (median, 193 pg ml$^{-1}$; 25th percentile, 163 pg ml$^{-1}$; 75th percentile, 253 pg ml$^{-1}$; controls) were found significantly elevated in all disconcerted melanoma patients (median, 281 pg ml$^{-1}$; 25th percentile, 208 pg ml$^{-1}$; 75th percentile, 307 pg ml$^{-1}$; $P = 0.031$), particularly in patients with distant melanoma metastasis (median, 285 pg ml$^{-1}$; 25th percentile, 239 pg ml$^{-1}$; 75th percentile, 363 pg ml$^{-1}$; $P = 0.012$). Plasma TGF-β2 in patients with locoregional involvement did not differ statistically from the control values (see Figure 1). The plasma levels of TGF-β3 were found unaltered in melanoma patients (median, 290 pg ml$^{-1}$; 25th percentile, 0 pg ml$^{-1}$; 75th percentile, 847 pg ml$^{-1}$) vs controls (median, 189 pg ml$^{-1}$; 25th percentile, 0 pg ml$^{-1}$; 75th percentile, 824 pg ml$^{-1}$; $P = 0.619$), irrespective of the disease stage.

### DISCUSSION

The source of elevated plasma levels of TGF-β1 and TGF-β2 is possibly tumour derived. TGF-β1 was shown to be the major secreted isoform in vitro, although in some cases TGF-β3 was found; small amounts of TGF-β2 were specifically secreted by melanoma cells and not by benign melanocytes (Krasagakis et al, 1997). Furthermore, increased in situ expression of TGF-β1, -β2, and -β3 has been reported in melanoma lesions during melanoma progression (van Belle et al, 1996), suggesting that these circulating factors are tumour derived and probably accumulate as a consequence of the increased tumour load in advanced disease stages.

The present findings support the hypothesis that elevated TGF-β1 and TGF-β2 plasma levels do correlate with advanced melanoma progression and may be indicative for the unfavourable prognosis at this tumour stage. Elevated levels of TGF-β1 have been reported in patients with other types of neoplasia, including colorectal and prostatic carcinoma, indicating disease progression (Ivanovic et al, 1995; Tsushima et al, 1996). The present data show for the first time elevated TGF-β peptide levels in melanoma patients, and also that, besides TGF-β1, other TGF-β isoforms such as TGF-β2 may be increased in plasma of cancer patients. Elevated levels of TGF-β1 and TGF-β2 may reflect or, because of the potent immunosuppressive properties of TGF-β, be even

© Cancer Research Campaign 1998 

causatively involved in the impairment of the immune system in melanoma patients with advanced tumour dissemination. Indeed, several lines of evidence suggest a role for tumour-derived TGF-β-mediated immunosuppression. A highly immunogenic tumour transfected with a murine TGF-β1 cDNA escaped immune surveillance in mice, and TGF-β2 has been identified as the T-cell suppressor factor of human glioblastoma (de Martin et al, 1987; Torre-Amione et al, 1990). Furthermore, treatment of mice with anti-TGF-β antibodies inhibited growth of human breast cancer cells by enhancing spleen NK-cell activity (Arteaga et al, 1993). In addition, B16 melanoma growth and metastasis in vivo was inhibited by treatment of mice with anti-transforming growth factor beta antibody and interleukin 2 (Wojtowicz-Praga et al, 1996), indicating that therapeutic interventions for blocking systemic TGF-β overexpression should be taken under further consideration.

### REFERENCES

Arteaga CL, Hurd SD, Winnier AR, Johnson MD, Fendly BM and Forbes JT (1993) Anti-transforming growth factor (TGF)-β antibodies inhibit breast cancer cell tumorigenicity and increase mouse spleen natural killer cell activity: implications for a possible role of tumor cell/host TGF-β interactions in human breast cancer cell progression. *J Clin Invest* **92**: 2569–2576

de Martin R, Haendler B, Hofer-Warbinek R, Gaugitsch H, Wrann M, Schlüsener H, Seifert JM, Bodmer S, Fontana A and Hofer E (1987) Complementary DNA for human glioblastoma-derived T cell suppressor factor, a novel member of the transforming growth factor-β gene family. *EMBO J* **6**: 3673–3678.

Ivanovic V, Melman A, Davis-Joseph B, Valcic M and Geliebter J (1995) Elevated plasma levels of TGF-β1 in patients with invasive prostate cancer. *Nature Med* **1**: 282–283

Krasagakis K, Garbe C, Schrier PI and Orfanos CE (1994) Paracrine and autocrine regulation of human melanocyte and melanoma cell growth by transforming growth factor beta in vitro. *Anticancer Res* **14**: 2565–2572

Krasagakis K, Krüger-Krasagakes S, Thölke D, Eberle J, von der Ohe M and Orfanos CE (1997) Increased synthesis of TGF-β isoforms (β1, β2, β3) by malignant melanoma cells occurs in vitro but does not inhibit their growth (abstract). *Arch Dermatol Res* **289S**: 28A

Luscher U, Filgueira L, Juretic A, Zuber M, Luscher NJ, Heberer M and Spagnoli GC (1994) The pattern of cytokine gene expression in freshly excised human metastatic melanoma suggests a state of reversible anergy of tumor-infiltrating lymphocytes. *Int J Cancer* **57**: 612–619

MacDougall JR, Kobayashi H and Kerbel RS (1993) Responsiveness of normal, dysplastic melanocytes, and melanoma cells from different lesional stages of disease progression to the growth inhibitory effects of TGF-β. *Mol Cell Diff* **1**: 21–40

Massague J (1990) The transforming growth factor-β family. *Annu Rev Cell Biol* **6**: 597–641

Reed JA, McNutt MS, Prieto VG and Albino AP (1994) Expression of transforming growth factor-beta 2 in malignant melanoma correlates with the depth of tumor invasion. Implications for tumor progression. *Am J Pathol* **145**: 97–104

Rodeck U, Bossler A, Graeven U, Fox FE, Nowell PC, Knabbe C and Kari C (1994) Transforming growth factor beta production and responsiveness in normal human melanocytes and melanoma cells. *Cancer Res* **54**: 575–581

Torre-Amione G, Beauchamp RD, Koeppen H, Park BH, Schreiber H, Moses HL and Rowley DA (1990) A highly immunogenic tumor transfected with a murine transforming growth factor type β1 cDNA escapes immune surveillance. *Proc Natl Acad Sci USA* **87**: 1486–1490

Tsushima H, Kawata S, Tamura S, Ito N, Shirai Y, Kiso S, Imai Y, Shimomukai H, Nomura Y, Matsuda Y and Matsuzawa Y (1996) High levels of transforming growth factor-β1 in patients with colorectal cancer: Association with disease progression. *Gastroenterology* **110**: 375–382

Van Belle P, Rodeck U, Nuamah I, Halpern AC and Elder DE (1996) Melanoma-associated expression of transforming growth factor-β isoforms. *Am J Pathol* **148**: 1887–1894

Wahl SM, McCartney-Francis N and Mehrenhagen S (1989) Inflammatory and immunomodulatory roles of TGF-β. *Immunol Today* **10**: 258–262

Wojtowicz-Praga S, Verma UM, Wakefield L, Esteban JM, Hartmann D and Mazumder A (1996) Modulation of B16 melanoma growth and metastasis by anti-transforming growth factor beta antibody and interleukin-2. *J Immunother Emphasis Tumor Immunol* **19**: 169–175

© Cancer Research Campaign 1998